# Efficacy of omalizumab in food allergic adults - A retrospective analysis

**DOI:** 10.1016/j.waojou.2025.101048

**Published:** 2025-04-03

**Authors:** Aikaterina Alexiou, Sofia Carreras-Kàtcheff, Karin Hartmann, Regina Treudler, Paolo Tassinari, Victoria Cardona, Margitta Worm

**Affiliations:** a Division of Allergy and Immunology, Department of Dermatology, Venerology and Allergy, Charité Universitätsmedizin Berlin, Germany; b Department of Allergy, Hospital Vall d'Hebron, Barcelona, Spain; c Division of Allergy, Department of Dermatology, University Hospital Basel and University of Basel, Basel, Switzerland; d Department of Biomedicine, University Hospital Basel and University of Basel, Basel, Switzerland; e Department of Dermatology, Venereology and Allergology, Leipzig Interdisciplinary Center for Allergology - LICA-CAC, University of Leipzig, Germany; f Institute of Allergology, Charité – Universitätsmedizin Berlin, Germany; g Novartis Pharma AG, Asklepios 8 – 6th Floor, 4002 Basel, Switzerland

**Keywords:** Food allergy, Omalizumab, Immunotherapy, Anaphylaxis

## Abstract

**Background:**

IgE-mediated food allergy poses a significant public health concern, currently with no approved therapies for adults in Europe. Omalizumab (OMA) used as monotherapy or in conjunction with oral immunotherapy (OIT) has been suggested as an efficacious treatment for severe food allergy. The aim of this study was to analyze real-world data from food-allergic patients treated with OMA.

**Methods:**

We included food-allergic patients treated with OMA between 2002 and 2022 throughout Europe. Treatment responders (TR) were identified based on the unresponsiveness to related food allergens (determined by food challenge), reduction in the severity of food allergy and absence of anaphylactic reactions.

**Results:**

Sixty-two patients (female n = 39/62, 62.9%; mean age 30.6 years) were included into this analysis, most of whom were polysensitized to more than 2 food allergens (n = 40/62, 64.5%); 45/62 patients (72.6%) received OMA in conjunction with OIT, while the remaining patients underwent OMA monotherapy. The eliciting food allergens were tree nuts (n = 27/62, 43.5%), cow's milk (n = 26/62, 41.9%), and vegetables (n = 25/62, 40.3%). In most cases, OMA was initiated with 300 mg q4w (n = 51/62, 82.3%) dosing. Treatment was tolerated exceptionally well.

Fifty-two (52/62) patients (83.9%) were classified as treatment responders. Six (6/62) patients (9.7%) developed unresponsiveness, 6/62 (9.7%) had a reduction of the severity of food allergy, and 40/62 (64.5%) had no further anaphylactic reactions during treatment. One (1/62) patient (1.6%) undergoing monotherapy was a non-responder, exhibiting repeated anaphylactic reactions to accidental exposures, and 10/62 patients (16.1%) reported anaphylactic reactions during treatment. In most of these cases, cofactors (n = 5/10, 50%) were present.

**Conclusion:**

Our real-world evidence data indicate efficacy and tolerability of OMA for the treatment of IgE-mediated food allergy with and without OIT. As the onset of food related reactions upon treatment was frequently linked to the presence of cofactors, these should be identified and considered in patients with food allergy—not only for diagnosis, but also in treatment settings.

## Introduction

Food allergies pose a significant public health concern, with their prevalence varying across regions and showing an increase in some countries in recent years.^1^^,^^2^

The underlying pathophysiological mechanism of food allergy is a type I immunologic mechanism. IgE is synthesized by B cells in response to allergen exposure and binds to IgE receptors (FcεRI and RII) via its Fc region on the surface membranes of various cells. Binding to FcεRI on mast cells and basophils is a key feature of the allergic effector phase.^3^ Upon allergen re-exposure cross-linking of receptor-bound IgE molecules occurs and results in cell activation and mediator release with the consecutive elicitation of symptoms.^4^ IgE perpetuates the allergic reaction by enhancing the expression of FcεRI on mast cells and basophils.^5^

Currently, the standard of care of food allergy includes the short-term management of acute reactions and long-term strategies to reduce the risk of further reactions.^1^^,^^2^ Besides the prescription of an emergency medication for patient self-use, the most important measure is the avoidance of the offending allergen in daily life. The latter one might be challenging for patients and their relatives and can severely limit the quality of life of the affected patients.^6^^,^^7^ As a long-term causal approach, oral immunotherapy (OIT) is used in many countries on a patient-based approach worldwide as no approved therapy for the treatment of food allergy in adults is exists. The only licensed OIT preparation in the United States and Europe is Palforzia™, which is a standardised OIT product for the treatment of peanut allergy in children.^8^

On the other hand, omalizumab (OMA) is a humanized monoclonal anti-IgE antibody, which has been approved for the treatment of allergic asthma, chronic spontaneous urticaria and nasal polyposis for many years. OMA exerts its action by binding to circulating IgE, reducing IgE receptor expression, and decreasing mediator release from mast cells and basophils.^9^ OMA has been successfully used in numerous studies and case reports in the treatment of food and insect venom allergy, can be used as monotherapy or as adjuvant in immunotherapy for the treatment of anaphylaxis.^10^, ^11^, ^12^, ^13^, ^14^, ^15^ Currently, patients with food or insect venom anaphylaxis can only be treated in an off-label setting with OMA if not suffering from a coexisting chronic spontaneous urticarial and/or severe asthma. Very recently, based on the positive outcome of the OUtMATCH study showing efficacy of OMA in food allergic children,^16^ OMA has been approved by the Food and Drug Administration in the United States for the treatment of food allergy.

While the combination of OMA and OIT has been explored in food allergies such as peanut and milk, no studies specifically address its application for lipid transfer protein (LTP) allergies. LTP allergies are increasingly recognized as a cause of severe allergic reactions, especially in Mediterranean populations. Managing these allergies poses a unique challenge due to the high risk of anaphylaxis and the complex nature of LTP proteins, which are often resistant to heat and digestion, making avoidance strategies insufficient.^17^

In this retrospective analysis, we aim to analyze real-life data from patients who have received OMA for the treatment of food allergy and food-induced anaphylaxis in different European countries.

## Methods

Patients who received therapy with OMA for the treatment of their food allergy between 2002 and 2022 were included into this retrospective analysis. Cooperative centers from the Anaphylaxis Registry were contacted to identify eligible participants and food allergic patients treated with OMA between 2002 and 2022 throughout Europe (Barcelona, Spain; Berlin and Leipzig, Germany; and Basel, Switzerland) were included. The assessment of the clinical data for research was approved by the ethics committee at the Charité (EA4/037/23) as the initiating center. In addition, local ethical approval was obtained in each center individually.

A structured survey questionnaire was set up for the retrospective data collection from the patient records, ensuring anonymization. The data obtained were analyzed descriptively. The questionnaire covered various parameters including demographics (sex, race, and year of birth), medical history (including relevant diseases, procedures, and medications), food allergy history (detailing specific allergies and anaphylactic reactions), diagnostic results (allergy tests and oral food challenges), OMA treatment details (including type, dosage, and safety), and information on oral immunotherapy for food allergy. Laboratory test results (total IgE, specific IgE, and basal serum tryptase) were obtained prior to treatment initiation.

Individuals were classified as treatment responders if an oral food challenge was negative (Responders- Group A), or a decrease of severity of food allergy during an oral food challenge was determined (Responders- Group B) and no anaphylactic reactions occurred during treatment (Responders- Group C). Non-responders were those who experienced repetitive food anaphylactic reactions during treatment. Partial treatment responders were defined as individuals who experienced less than 1 food anaphylactic reaction despite undergoing treatment.

The data were collected and entered into an Excel database. Analysis was conducted using IBM SPSS Statistics (version 27, Chicago, Ill).

## Results

### Demographics & food allergy history

In total, 62 patients were included into this analysis. Of those, 52 were treated in the allergy department at Hospital Vall d'Hebron in Barcelona, Spain, 5 in the allergy department at Charité Universitätsmedizin in Berlin, Germany, 4 in the allergy department at the University of Leipzig, Germany, and 1 at the allergy department at the University Hospital Basel, Basel, Switzerland. Most patients were female (n = 39/62, 62.9%), and all patients were Caucasian (n = 62/62, 100%). The age range of the patients was 9–59 years, with a mean age of 30.6 years at treatment initiation (Table 1).

**Table 1:** Demographics

	Entire cohort N = 62	Spain N = 52	Germany/Switzerland N = 10
**Age in years**			
Mean age	30.6	29.4	37.4
Median age	27	25.5	35
Min.-max. age	9–59	9–59	19–58
**Gender: n (%)**			
Female	39 (62.9%)	33 (63.5%)	6 (60%)
**Atopic history: n (%)**			
Food allergy	62 (100%)	52 (100%)	10 (100%)
Allergic rhinoconjuctivitis	43 (69.4%)	37 (71.1%)	6 (60%)
Asthma	34 (54.8%)	28 (53.8%)	6 (60%)
Atopic dermatitis	7 (11.3%)	5 (9.6%)	2 (20%)
Chronic spontaneous urticaria	5 (8%)	2 (3.8%)	3 (30%)
**IgE levels (kU/l)**			
Mean IgE levels (kU/l)	606.6	635.1	435.3
Median IgE levels (kU/l)	289	289	361
**Tryptase levels (μg/l)**			
Mean tryptase levels (μg/l)	4.4	4.4	4.7
Median tryptase levels (μg/l)	3.97	3.69	4.51

Medical history data covered atopic and other related diseases. The history of all patients included food allergy (n = 62/62, 100%), with the majority also presenting with allergic rhinoconjunctivitis (n = 43/62, 69.4%) and asthma (n = 34/62, 54.8%). Food allergy diagnosis was confirmed primarily through documentation of previous anaphylaxis at Grade 2 or higher, according to the Ring & Messmer anaphylaxis grading score (n = 52/62, 83.9%). Oral food challenges were conducted in 8 cases to confirm the diagnosis (n = 8/62, 12.9%), and in 2 cases, diagnosis was based on medical history alone (n = 2/62, 3.2%) due to oral allergy symptoms or urticaria. The median total IgE level was 289 kU/l and the median tryptase level was 3,97 μg/l at treatment initiation (Table 1). The majority of patients (n = 58/62, 93.5%) had a positive skin prick test to the culprit food (native or commercial extract) and/or their main components (commercial extracts).

**Fig. 1:**
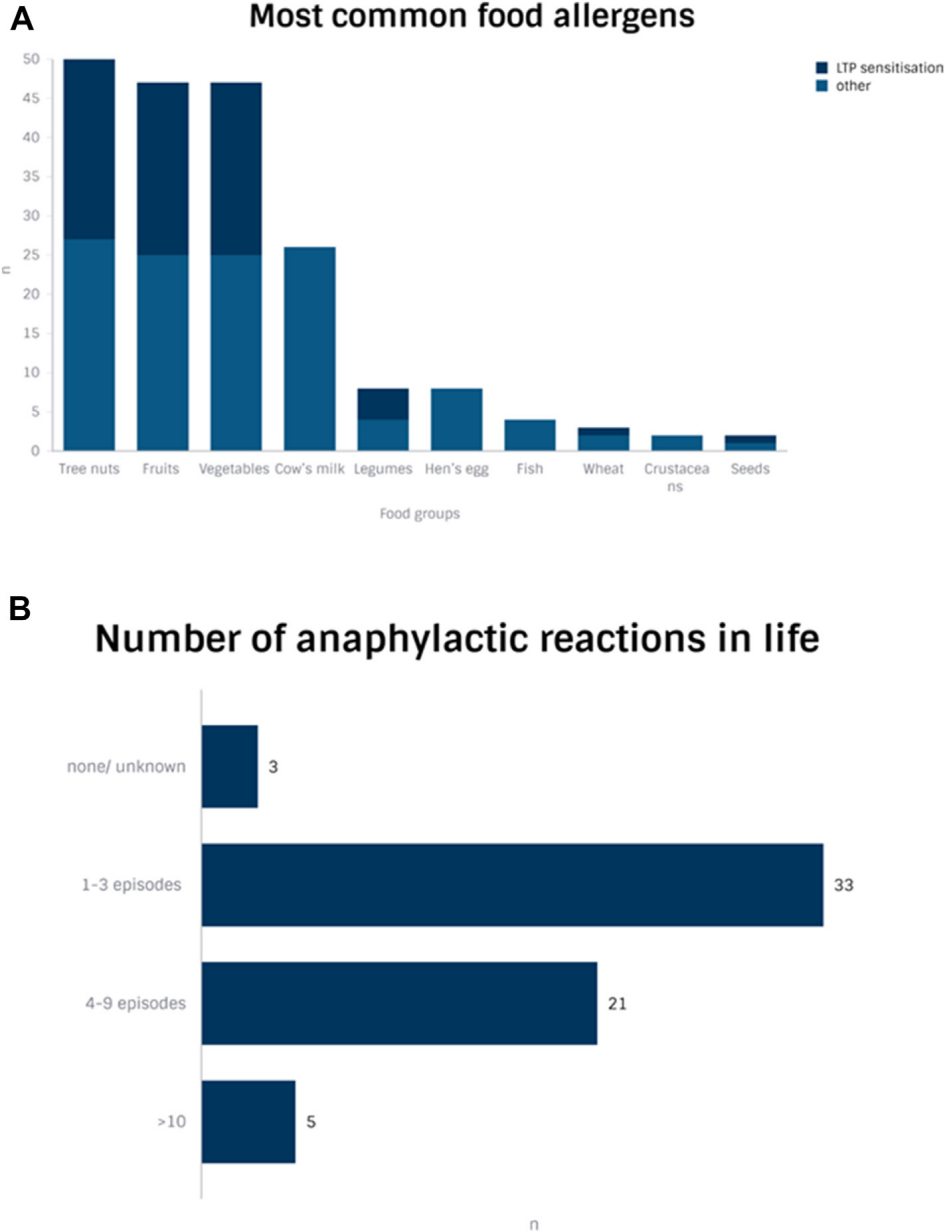
A depicts the eliciting food allergens by frequency. The proportion of cases with LTP sensitization is marked in dark blue. B depicts the number of anaphylactic reactions per case

Many patients (n = 40/62, 64.5%) were polysensitized to more than 2 food allergens. The eliciting food allergens were most frequently tree nuts (n = 27/62, 43.5%), cow's milk (n = 26/62, 41.9%), fruits (n = 25/62, 40.3%), and vegetables (n = 25/62, 40.3%) (Table 2, Fig. 1A). Furthermore, most of the patients underwent at least 1 episode of anaphylaxis before treatment, with n = 21/62 (33.9%) reporting 4 to 9 episodes, and 5/62 (8%) reporting at least 10 episodes of anaphylaxis in life (Fig. 1B).

**Table 2:** Food allergy history.

	Entire cohort N = 62	Spain N = 52	Germany/Switzerland N = 10
**Poly- VS monosensitised: n (%)**			
Polysensitised	40 (64.5%)	34 (65.4%)	6 (60%)
Monosensitised	22 (35.5%)	18 (34.6%)	4 (40%)
**Diagnosis of food allergy: n (%)**			
Anaphylaxis^a^	52 (83.9%)	47 (90.4%)	5 (50%)
Oral food challenge^b^	8 (12.9%)	4 (7.7%)	4 (40%)
Medical history^c^	2 (3.2%)	1 (1.9%)	1 (10%)
**Most common food allergens: n (%)**			
Cow's milk	26 (41.9%)	21 (40.4%)	5 (50%)
Hen's egg	8 (12.9%)	8 (15.4%)	0 (0%)
Vegetables	25 (40.3%)	22 (42.3%)	3 (30%)
Fruits	25 (40.3%)	20 (38.5%)	5 (50%)
Legumes	4 (6.5%)	3 (5.8%)	1 (10%)
Tree nuts	27 (43.5%)	22 (42.3%)	5 (50%)
Seeds	1 (1.6%)	1 (1.9%)	0 (0%)
Fish	4 (6.5%)	4 (7.7%)	0 (0%)
Crustaceans	2 (3.2%)	2 (3.8%)	0 (0%)
Wheat	2 (3.2%)	1 (1.9%)	1 (10%)

a (≥Grade 2, based on Ring & Messmer anaphylaxis grading score).

b With or without history of previous anaphylaxis.

c Based on previous symptoms in medical history, including Oral allergic symptoms and/or urticaria

### Treatment

**Fig. 2:**
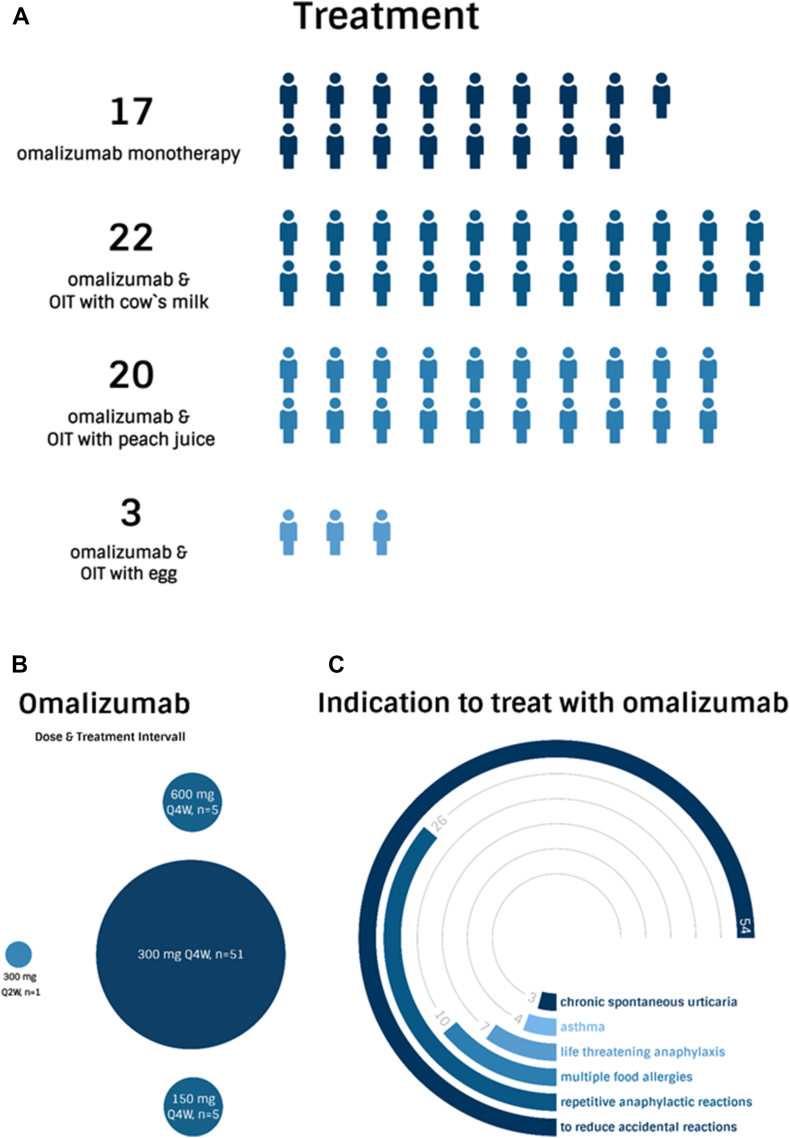
A shows the number of patients per treatment group. B shows the most common dose and treatment intervals used. C shows the indications for treatment with omalizumab

Seventeen individuals (n = 17/62, 27.4%) underwent OMA monotherapy, while the remaining 45 patients (n = 45/62, 72.6%) received OMA combined with oral immunotherapy (OIT). Among the latter group, OIT with cow's milk was performed in 22 cases (n = 22/62, 35.5%), OIT with peach juice for LTP allergy in 20 cases (n = 20/62, 32.6%) and OIT with egg in 3 cases (n = 3/62, 5%) (Fig. 2A). In the majority of cases, OMA was administered at treatment initiation with a dose of 300 mg subcutaneously every 4 weeks (n = 51/62, 82.3%) (Fig. 2B). The primary medical indication for the treatment was “to reduce an accidental reaction” (n = 54/62, 87%), followed by “onset of repetitive anaphylactic reactions” (n = 26/62, 41,9%) and “multiple food allergies” (n = 10/62, 16.1%) (Fig. 2C). The majority of the patients (n = 61/62, 98.4%) reported excellent safety with OMA treatment. One patient (n = 1/62, 1.6%) reported recurrent abdominal pain after receiving injections of OMA. Consequently, the treatment was discontinued due to this adverse event. The majority of patients (n = 48/62, 77.4%) still receive OMA with or without OIT, with a mean duration of treatment of 5 and one-half years and a median duration of treatment of 5 years. Twelve patients received OMA treatment for longer than 7 years. The remaining patients (n = 14/62, 22.6%) terminated the treatment. Seven of these patients continued the treatment with the OIT maintenance dose after OMA termination. The 7 patients who discontinued OMA are still avoiding the culprit foods.

### Outcome

**Fig. 3:**
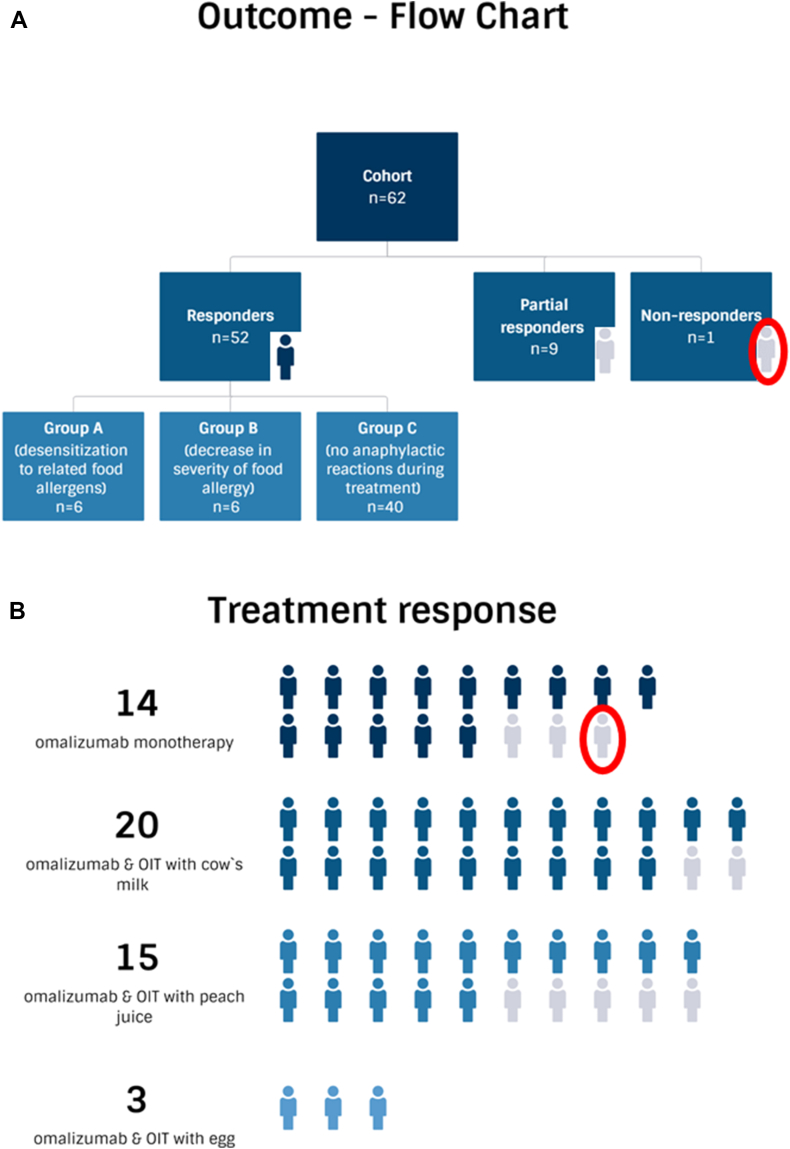
A is the outcome flowchart. B shows the treatment response per treatment group. Treatment responders are highlighted, partial responders are marked in grey, and non-responders are marked with a red circle

Fifty-two patients (n = 52/62, 83.9%) were classified as treatment responders. Among these, 6 cases (Group A, n = 6/62, 9.7%) achieved desensitization, which was confirmed by an oral food challenge. Group B (n = 6/62, 9.7%) consisted of patients who demonstrated a decrease in the severity of food allergy, either demonstrated by an oral food challenge or based on the clinical assessment by the investigator. Group C included patients who did not experience any anaphylactic reactions during treatment (n = 40/62, 64.5%). Nine patients (n = 9/62, 14.5%) were classified as partial responders due to experiencing anaphylactic reactions during treatment. One patient (n = 1/62, 1.6%) was categorized as a non-responder (Fig. 3A).

Analyzing treatment groups that received OMA monotherapy versus OMA with concomitant OIT, the clinical responses yielded the results that follow. Fourteen patients (n = 14/17, 82.4%) who underwent OMA monotherapy were classified as responders. Twenty patients (n = 20/22, 90.1%) who received OMA combined with OIT with cow's milk were characterized as responders, as well as 15 (n = 15/20, 75%) of those who received OMA with OIT with peach juice. Additionally, all 3 patients (n = 3/3, 100%) who received OMA combined with OIT with egg were characterized as responders (Fig. 3B).

### Food anaphylaxis during treatment

Ten patients (n = 10/62, 16.1%) reported anaphylactic reactions related to food and/or to OIT during treatment (Supplementary Table 1). Three of these patients received OMA monotherapy, while 2 received OMA combined with OIT with cow's milk, and the remaining 5 received OMA with OIT with peach juice (Fig. 3). In 5 cases, the reactions were OIT treatment related and related to the presence of cofactors such as exercise, non-steroidal anti-inflammatory drugs (NSAIDs), menstruation, and alcohol, and in 1 case, the reaction was related to OIT with cow's milk due to patient non-compliance with therapy. The treatment was prematurely terminated in 3 of these cases. One patient experienced repetitive severe anaphylactic reactions and was classified as a non-responder; this patient terminated the treatment after 18 months (Supplementary Table 1).

### Comparison of the groups: Milk-allergic patients & LTP-allergic patients

When comparing patients undergoing OIT for cow's milk and LTP allergies, the following differences were revealed. Twenty-six patients were diagnosed with cow's milk allergy, and 20 with LTP allergy. Diagnosis of LTP food allergy was established through a combination of clinical history indicating anaphylaxis to LTP-containing foods (eg, peach, apple, hazelnut) and specific IgE testing for LTP proteins, primarily for peach and/or Pru p 3.LTP-allergic patients had a higher age at treatment initiation, with a mean age of 36.3 years. Furthermore, cow's milk-allergic patients exhibited higher total IgE levels (mean total IgE levels: 1024 kU/l VS 288.3 ku/l) and higher specific IgE levels (mean cow's milk sIgE: 57 kU/l VS mean peach sIgE: 21.9 kU/l). The percentage of responders was higher among the cow's milk-allergic patients (90.1% VS 75%). However, treatment desensitization was similarly favorable in both groups (Table 3).

**Table 3:** Cow's milk allergy VS LTP allergy

	LTP allergy N = 20	Cow's milk allergy N = 26
**Age in years**		
Mean age	36.3	18.2
**IgE levels (kU/l)**		
Mean total IgE levels (kU/l)	288.3	1024
Mean cow's milk sIgE levels (kU/l)	n.a.	57
Mean peach sIgE levels (kU/l)	21.9	n.a.
Mean pru p3 levels (kU/l)	16.5	n.a.
Mean a-lactalbumin levels (kU/l)	n.a.	29.9
Mean b-lactalbumin levels (kU/l)	n.a.	14.1
Mean casein levels (kU/l)	n.a.	70.8
**Treatment tolerance**		
Treatment tolerated well	20/20 (100%)	26/26 (100%)
**Outcome**		
Responders	15/20 (75%)	22/26 (84.6%)
Partial responders	5/20 (25%)	3/26 (11.5%)
Non responders	0/20 (0%)	1/26 (3.8%)

## Discussion

In the present study, we analyzed real-world data on treatment of food allergy with OMA from various countries, encompassing patients with different sensitization patterns and subjected to various treatment protocols. This study involved a substantial cohort of adult patients throughout Europe and highlights the significant efficacy of OMA in managing food allergies.

There is currently great scientific interest in the field of biologics used in food allergies. Overall, our data provide real-world evidence for the efficacy and tolerability of OMA in IgE-dependent food allergy with and without OIT, aligning with existing literature.^15^^,^^16^^,^^18^ To our knowledge, our study is the first to publish the use of OMA in combination with OIT with peach juice for patients with proven LTP sensitization and highlights that OMA also facilitates OIT in patients with LTP allergy.

In comparison with the 2 recently published studies that included mainly children who underwent monotherapy with OMA for a short time period of 3 to 4 months,^16^^,^^18^ we could demonstrate the challenges facing the treatment of adult patients with food allergies. The aforementioned studies followed the total IgE-adjusted doses recommended for asthma, while our cohort mainly underwent an individually dosed concept, which has also been proven to be effective.^19^^,^^20^ Our findings also highlight the excellent tolerability of OMA over a larger period of time, considering that our data also included patients who had been under treatment with OMA for over 9 years.

In 82.3% of cases, OMA was administered at a dose of 300 mg every 4 weeks, consistent with the dose approved for chronic spontaneous urticaria. This dosage was not predetermined but was adopted across various European allergy centers based on clinical experience, as it is also practical to implement. However, literature suggests that individualized OMA dosing may offer a more effective and safer treatment for severe food allergies, such as peanut allergy.^19^ These studies also suggest that the specific IgE to storage proteins could predict the need for a higher omalizumab dose.^19^ In the OUtMATCH study, the OMA dose and frequency were based on patient weight and total IgE levels.^16^ Further research is necessary to establish optimal dosing, and specific IgE to the culprit allergens may be a more accurate marker for predicting the optimal dose.

Notably, our study highlights a significant proportion of patients sensitized to LTP, which can likely be attributed to regional variations in sensitization patterns.^17^ In contrast, this group comprised only a small proportion of patients allergic to storage proteins, a finding that was observed in other studies who resulted in reduced recruitment of adults.^8^^,^^16^ This latest observation also highlights the absence of standardized protocols for OIT for peanut or other allergies, as well as the elevated risk environment associated with such allergies. Another interesting observation in our study were the clinical differences observed between LTP and milk allergic patients. Notably, LTP-sensitized patients had a higher age and experienced an increased incidence of anaphylactic reactions during treatment. Whether this trend may be related to the cross-reactivity among LTP-related food allergens requires further exploration.

In most cases, anaphylaxis was not present during treatment. Interestingly, the onset of recurrent reactions upon treatment in single patients was linked to the presence of cofactors. Such cofactors were exercise, NSAIDs, menstruation, and alcohol. The presence of cofactors and their role in anaphylaxis and reaction severity was proven several times.^21^^,^^22^ Cofactors seem to be the remaining obstacle in the management of food allergy. Therefore, we propose that cofactors should not only be identified and considered in adult patients with food allergy for diagnosis, but also in respect to treatment.

Our study has several limitations. First, the data collection method, relying on paper-based questionnaires and a combination of closed and open questions, may introduce variability and subjectivity into the findings. Additionally, the lack of quality of life (QoL) data collected in real-life settings is another limitation. Moreover, the lack and/or non-standardised oral food challenge (OFC) data, which is crucial for the risk assessment of the allergic reactions, complicates the outcome evaluation. Given that this study was limited to a few centers in Europe, the generalizability of our findings is limited.

Furthermore, as a retrospective study, our findings may be subject to selection bias due to the reliance on previously documented cases, which may not fully represent the broader patient population. Future studies could mitigate this by adopting a prospective design with controlled selection criteria and incorporating randomized sampling or multi-center data sources for a more representative sample.

Despite its limitations, our study offers valuable insights into how disparities across European healthcare systems and allergology practices impact access to allergy treatments. Significant differences between European countries exist not only in healthcare funding and reimbursement policies but also in the recognition and scope of allergology as a medical specialty. For instance, Spain has recognized allergy as an independent specialty for over 4 decades, providing clearly defined roles and competences, unlike other European countries where allergy may be classified as a subspecialty within ENT or dermatology, affecting patient access to specialized care. Additionally, sensitization patterns vary between central and southern Europe,^23^ influenced by environmental exposures and genetic factors. These regional differences extend to treatment approaches, such as the preference for OIT versus monotherapy, shaped by local clinical guidelines, resources, reimbursement structures, and patient preferences. Together, these findings underscore the need for standardized policies to ensure equitable access to allergy diagnostics and treatments across Europe, thus enhancing patient outcomes and quality of life.

In conclusion, our data suggest the efficacy and tolerability of OMA in treating IgE-mediated food allergy, both alone and in combination with OIT. Notably, our study shows that OMA, particularly when combined with OIT, achieves higher response rates. Regional variations in sensitization patterns underscore the need for tailored allergy treatment across populations. The data further support OMA and OIT as viable long-term options with good safety and patient adherence, though ongoing research is essential to refine these approaches, especially for patients with complex allergy profiles. Recurrent reactions during treatment were often linked to the presence of cofactors, which should therefore be identified and managed in both diagnostic and treatment settings for food allergy.

## Abbreviations

ENT: ear nose & throat doctor/ otorhinolaryngologist; IgE: Immunoglobulin E; LTP: lipid transfer protein; NSAIDs: non-steroidal anti-inflammatory drugs; OFC: oral food challenge; OIT: oral immunotherapy; OMA: Omalizumab; QoL: quality of life; TR: treatment responder.

## Author contributions

All authors contributed to patients care and supported data collection. AA, RT, SCK performed data collection. AA and SCK performed literature research. AA performed statistical analysis and data interpretation and wrote the manuscript. MW conceived the study design, coordinated the manuscript and the data analysis. All authors reviewed and approved the final manuscript.

## Funding information

A. Alexiou is funded by the Deutsche Forschungsgemeinschaft (DFG, German Research Foundation) as part of the clinical research unit (CRU339): Food allergy and tolerance (FOOD@) – project-number 428447634.

## Declarations of competing interest

AA is a sub investigator in several clinical studies sponsored by pharma including Novartis. SCK and VC have no conflicts of interest to declare. KH and RT received honoraria for lectures, advisory boards and/or research funding from Novartis and other pharmaceutical companies, all outside this research project. MW received compensation for research, advisory and speaker from Novartis and other pharmaceutical companies.
